# Abstracts DGMKG

**DOI:** 10.1515/iss-2019-2005

**Published:** 2019-03-20

## DGMKG/DGPR-C: Post-operative management in patients with microvascular transplants

### Impact of intraoperative use of vasopressors in lower extremity reconstruction: single centre analysis of 437 free gracilis muscle and fasciocutaneous anterolateral thigh flaps

(Abstract ID: 76)

P. Heidekrueger^1^, L. Prantl^1^, A. Thiha^1^, M. Ninkovic^2^, N. Broer^2^

^1^*Universitätsklinikum Regensburg*

^2^*Klinikum Bogenhausen, München*

**Background:**

While complication rates in free tissue transfers have continuously decreased over time due to improved techniques, the intraoperative use of vasopressors and their negative effects on flap microcirculation and patency of the anastomoses remains controversial. To further elucidate this matter, this retrospective study examines the effect of intraoperative vasopressors on free gracilis muscle and free fasciocutaneous anterolateral thigh (ALT) flaps for lower extremity reconstruction.

**Materials and methods:**

Over a six year period, 425 patients underwent 437 free flaps for lower limb reconstruction at a single surgical centre. The series was divided into two groups: use of intraoperative vasopressors (V; n=318) or no use (NV, n=119). The data were retrospectively screened for patients’ demographics, perioperative details, and surgical complications.

**Results:**

The two groups were comparable regarding patient comorbidities. There were no significant difference between the groups regarding major complications, i.e. total flap loss (V: 5.35 percent (17/318) versus NV: 5.04 percent (6/119), p=0.899) or revision rate (V: 18.87 percent (60/318) versus NV: 12.61 percent (15/119); p=0.122), or minor complications, i.e. partial flap loss (V: 6.29 percent (20/318) versus NV: 5.88 percent (7/119),p=0.875). Further, there were no significant differences between the types of flap used (free gracilis or ALT flap).

**Conclusion:**

This study confirms that the usage of intraoperative vasopressors has no influence on free flap survival rate in lower extremity reconstruction. It seems to be no difference between free muscle or fasciocutaneous flaps. Over the last decade vasopressors have therefore been routinely used in our clinic to overcome perioperative hypotension.

